# Acral Amelanotic Melanoma With a Sporotrichoid Pattern and Associated Vitiligo in an Elderly Patient: A Case Report

**DOI:** 10.7759/cureus.88630

**Published:** 2025-07-23

**Authors:** Ximena Rodríguez-Rangel, Alexa María Flores-Leonel, Marianne Signoret-Bravo, Sonia Toussaint-Caire, Ana L Ramirez Teran

**Affiliations:** 1 Dermatology, Hospital General "Dr. Manuel Gea González", Mexico City, MEX; 2 Dermatopathology, Hospital General "Dr. Manuel Gea González", Mexico City, MEX

**Keywords:** acral amelanotic melanoma, nonsegmental vitiligo, older adult, prognostic, sporotrichoid

## Abstract

Acral amelanotic melanoma (AM) is a rare and aggressive melanoma subtype that frequently presents with a diagnostic delay due to its atypical characteristics. Sporotrichoid spread, suggesting lymphatic dissemination, is rarely seen. Vitiligo, although usually benign, may signal underlying malignancy when appearing de novo in older adults. An 82-year-old woman presented with an 18-month history of a foul-smelling, ulcerated nodular lesion on the left foot, accompanied by multiple erythematous nodules in a linear pattern ascending to the ipsilateral groin. She also had achromic macules consistent with non-segmental vitiligo, present for two years. A biopsy revealed invasive nodular AM (Breslow ≥4 mm, Clark level IV) with perineural and vascular invasion. She was referred for oncologic evaluation and treatment. This case highlights a rare clinical presentation of acral AM with sporotrichoid spread and associated vitiligo in an elderly patient. Given its frequent misdiagnosis, clinicians should consider AM in atypical, nonresponsive lesions, particularly when associated with late-onset vitiligo.

## Introduction

Amelanotic melanoma (AM) is an uncommon and diagnostically challenging variant of cutaneous melanoma, characterized by the absence or minimal presence of melanin pigmentation. This lack of pigmentation frequently leads to misdiagnosis, as lesions may clinically mimic benign or inflammatory conditions such as eczema, warts, granulomatous infections, or non-melanocytic neoplasms. AM typically comprises 2-8% of all melanomas and is associated with a worse prognosis due to delayed detection and more advanced stages at diagnosis [1].

Melanoma exhibits significant epidemiologic variability across populations. In fair-skinned individuals, the superficial spreading subtype is predominant, whereas in populations with darker skin tones, such as in Latin America and Asia, acral lentiginous melanoma (ALM) is more prevalent. In Mexico, ALM accounts for approximately 45% of all melanomas, often involving the soles, palms, and subungual areas. However, acral AM is particularly rare, with a reported prevalence of only 2-3% of all acral melanomas [2].

Clinically, melanoma is classified into several subtypes, including superficial spreading, nodular, lentigo maligna, and ALM, with each variant demonstrating distinct anatomic predilections and growth patterns [2]. Diagnosis frequently relies on immunohistochemical staining, with markers such as S-100, SOX10, Melan-A (MART-1), and HMB-45 being essential for confirmation [1,3].

An especially uncommon presentation of AM is with sporotrichoid spread, a pattern characterized by linear nodules tracking along lymphatic pathways. This distribution is typically associated with infections such as sporotrichosis, atypical mycobacteria, or nocardiosis, and its presence in melanoma is exceedingly rare, further obscuring early recognition and contributing to diagnostic delay [1].

In addition, vitiligo, an acquired depigmentation disorder, may in rare cases be associated with melanoma. Although generally benign, new-onset non-segmental vitiligo in older adults should prompt consideration of underlying melanoma, particularly when localized to areas near or distant from the primary tumor. This phenomenon is believed to reflect an immune-mediated response against melanocyte antigens shared by melanoma cells [4].

We present a rare case of acral AM with sporotrichoid lymphatic spread and concurrent non-segmental vitiligo, highlighting the diagnostic complexity of atypical melanoma variants and the importance of maintaining a high index of suspicion in unusual clinical contexts.

## Case presentation

**Figure 1:**
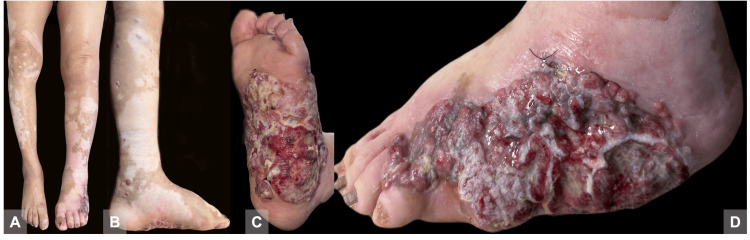
A. Vitiligo. B. Sporotrichoid pattern. C and D. Multiple ulcerated erythematous nodules.

An 82-year-old woman presented with an 18-month history of a painful, foul-smelling dermatosis on the left lower extremity, affecting the dorsum and sole of the foot. Physical examination revealed a 15 × 20 × 0.5 cm anfractuous lesion composed of multiple ulcerated erythematous nodules with slough and fibrin, indurated and exuding serohematic discharge. Ascending from the foot to the ipsilateral groin were several firm erythematous nodules distributed in a sporotrichoid pattern (Figure 1). The patient reported the lesion had developed following trauma to the plantar surface and had been unsuccessfully treated with antibiotics by multiple physicians. 

**Figure 2:**
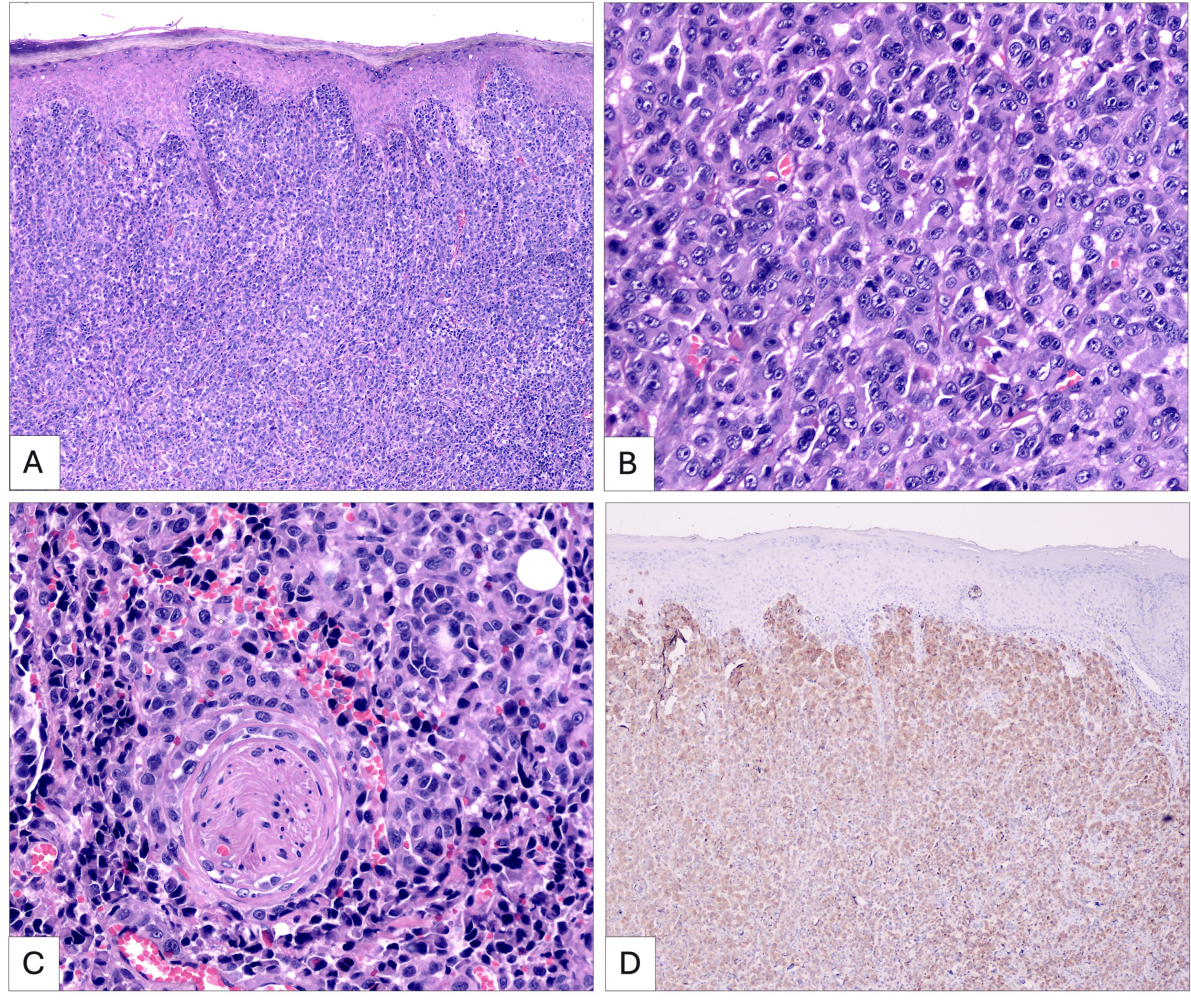
A. Histopathological microphotograph of invasive cutaneous melanoma showing a dense and diffuse proliferation of atypical melanocytes in the superficial and deep dermis. B. Closer histological view of the lesion, composed of large pleomorphic epithelioid-shaped melanocytes, with enlarged nucleoli, coarse chromatin, and mitotic figures. C. Evidence of perineural invasion, in which numerous atypical melanocytes have peripherally infiltrated a small nerve. D. Immunohistochemistry study with Melan-A marker is positive, showing intense, diffuse positive cytoplasmic labeling in proliferating melanocytes.

In addition, she presented with a two-year history of asymptomatic, well-demarcated achromic macules involving the entire body, consistent with non-segmental vitiligo. An incisional biopsy was performed with a clinical differential of sporotrichosis versus melanoma. Histopathological analysis revealed an invasive nodular melanoma composed of atypical melanocytes with vascular and perineural invasion, positive for Melan-A and CD31. The lesion had a Breslow thickness ≥4 mm and was classified as Clark level IV (Figure 2). The patient was referred to the oncology service for staging and treatment.

The patient was referred to the oncology service for staging and treatment.

## Discussion

The clinical presentation of acral AM represents a formidable diagnostic challenge due to its morphological variability and absence of pigmentation, often delaying detection until advanced stages where therapeutic options are limited. In our case, the significant Breslow depth and presence of ulceration, key determinants of the advanced pathological staging, contributed to the aggressive nature of the disease. Well-established indicators of a poor prognosis and increased metastatic risk include advanced patient age, acral location, and histopathological findings such as increased Breslow depth, ulceration, and a high mitotic index, particularly when compared to pigmented melanomas [1,4,5]. Given the complexity of its diagnosis and inherent aggressiveness, the literature underscores the critical importance of case reports detailing unusual clinical characteristics, such as those observed in our patient.

In the presented case, the sporotrichoid distribution, mimicking lymphocutaneous infections, was particularly striking. While this pattern has been sporadically reported in pigmented melanoma, its documentation in AMs is exceedingly rare and, to our knowledge, has not been previously described in the literature. This presentation likely reflects an atypical lymphatic dissemination. Kamble et al. described a similar case in a 50-year-old woman with sporotrichoid melanoma and distant metastases [6], highlighting the aggressive nature associated with this pattern. Our case, therefore, significantly contributes to the existing literature by being the first report of acral AM with a sporotrichoid dissemination pattern, offering a valuable addition to the spectrum of atypical presentations clinicians must consider.

The coexistence of vitiligo and melanoma, as observed in our patient, is an area of growing interest. Although vitiligo is typically a benign condition, its de novo appearance in older adults can serve as a red flag for underlying malignancy, including melanoma [7]. This association is postulated to stem from shared immunological mechanisms, where common antigens between melanocytes and melanoma cells may trigger an autoimmune response [4]. While the presence of immunotherapy-induced vitiligo in melanoma patients has been correlated with improved treatment response and prognosis, it is crucial to note that, in untreated patients such as ours, vitiligo does not appear to mitigate the inherently poor prognosis associated with acral AM. Furthermore, KIT mutations, frequently observed in acral melanomas, have been implicated in vitiligo pathogenesis, suggesting a potential shared molecular basis.

## Conclusions

Acral AM with sporotrichoid spread is a rare and aggressive presentation that can mimic benign or infectious conditions, delaying diagnosis and worsening prognosis. The presence of late-onset vitiligo in elderly patients should prompt thorough evaluation for possible malignancy; however, it is important to reiterate that while their co-occurrence warrants heightened clinical attention, direct causality between vitiligo and melanoma remains unconfirmed. This case underscores the importance of maintaining a high index of suspicion in chronic, atypical dermatoses and contributes to the literature on rare melanoma variants and their potential association with vitiligo.
